# Protein-Taggable Near-Infrared Photocages for Theranostics of Alzheimer’s Disease

**DOI:** 10.1021/acscentsci.5c00553

**Published:** 2025-04-07

**Authors:** Mengmeng Ma, Yanli Zhao

**Affiliations:** † School of Chemistry, Chemical Engineering and Biotechnology, 54761 Nanyang Technological University, 21 Nanyang Link, Singapore 637371, Singapore

## Abstract

Protein-tagged near-infrared-activatable photocages have been developed for the simultaneous diagnosis and treatment of Alzheimer’s disease.

## Graphical abstract

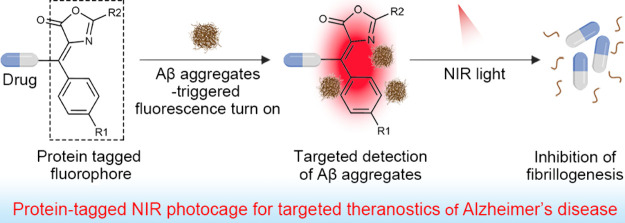

Photoactivatable protecting groups (PPGs) are beneficial tools for spatiotemporally controlled release of enzymes, fluorophores, signaling molecules, and neurotransmitters, proving invaluable to biological chemistry and medicine.^1,2^ Photoactivation in biological systems imposes specific requirements on PPGs, with three critical aspects being particularly important. (1) Release kinetics: The PPGs must demonstrate a sufficiently high release rate to avoid any interference of the release step with dynamic processes in chemical biology or the action of drugs. (2) Aqueous compatibility: PPGs must function in water and produce nontoxic byproducts. (3) Near-infrared (NIR) activation: On account of the reduced toxicity, deep tissue penetration, and minimal interference from endogenous biomolecules such as hemoglobin and melanin, NIR light has become an attractive stimulus for developing PPGs. These criteria highlight the need for PPGs with fast release kinetics, water compatibility, and NIR activation in effective biological applications.

To meet these requirements, various NIR light-activated photocages, including BODIPY and cyanine dyes, have been designed and prepared to explore various biological processes. However, these photocages lack inherent protein tagging capabilities, thus hindering their biomedical applications.

Alzheimer’s disease (AD), affecting ∼55 million people globally, poses a major socio-economic burden.^3^ At present, the clinical gold standard for AD diagnosis primarily relies on positron emission tomography imaging or cerebrospinal fluid analysis of amyloid-β (Aβ).^4^ However, these approaches remain constrained by major practical barriers: high operational complexity, substantial costs, and dependence on specialized instrumentation and expertise, rendering them largely impractical for resource-limited settings. Recent advances in molecular designs have introduced innovative diagnostic approaches in AD. Notably, numerous fluorescent probes have been developed over the past 20 years for Aβ detection in early AD diagnosis.^5^ Nevertheless, a critical unmet need persists: current probes predominantly serve diagnostic purposes, while overlooking their therapeutic potential to disrupt fibrillogenesis.

In this issue of *ACS Central Science*, Singh, Guha, Kim, and co-workers report a new class of protein-tagged NIR-activatable photocages based on green fluorescent protein (GFP) chromophores (namely meso-GFP-PRPG), enabling simultaneous protein tagging and controlled bioactive release, for targeted AD theranostics.

By demonstrating the theranostic potential, the optimal photocage, i.e., 15E, released valproic acid, a neuroprotective agent, upon 640 nm irradiation, effectively degrading Aβ aggregates implicated in AD.^1^ Notably, 15E exhibited strong NIR emission upon binding to Aβ oligomers and fibrils, achieving a 30–37-fold fluorescence enhancement over thioflavin T (ThT), with a detection limit of 1.2 pM. This breakthrough establishes 15E as a versatile theranostic platform that achieves Aβ detection and Aβ disaggregation (Figure 1), holding great promise for the theranostics of AD. Overall, this work bridges a critical gap in photochemistry by introducing protein-tagged photoactivatable photocages for AD treatment. The developed photocage, 15E, serves as a theranostic agent for the simultaneous diagnosis and treatment of AD.

**Figure 1:**
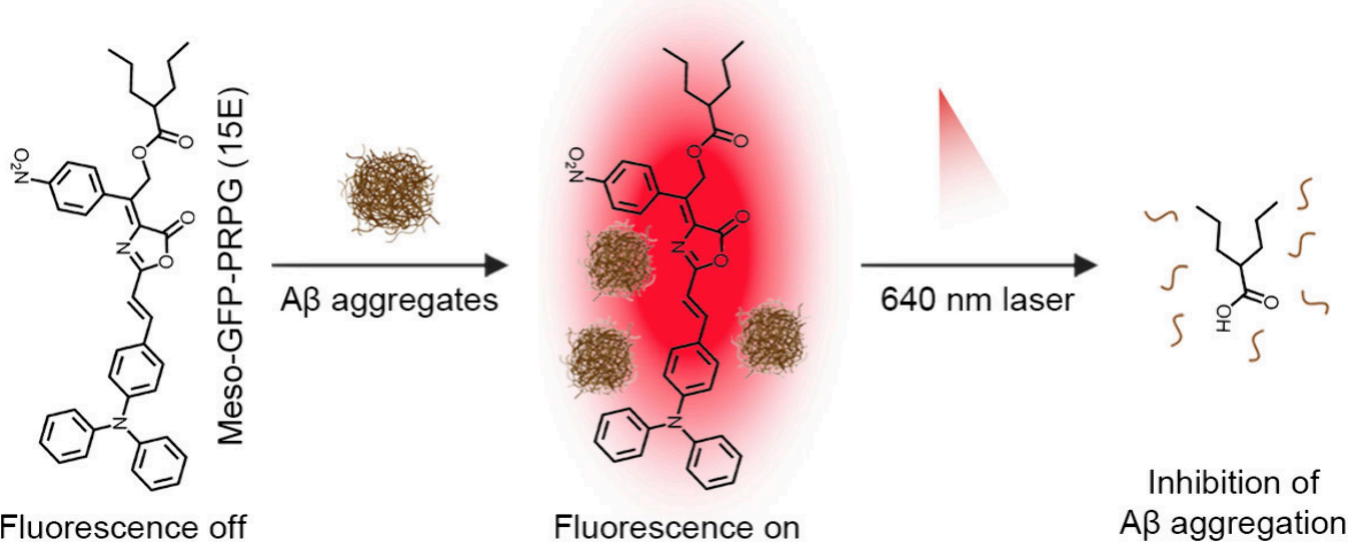
Protein-tagged NIR-activatable photocage meso-GFP-PRPG (15E) for targeted detection of Aβ and prevention of Aβ aggregation.

Beyond AD, the protein-tagged photoactivatable photocages could be extended to other protein-misfolding disorders, such as Parkinson’s and Huntington’s diseases, offering a versatile approach for early diagnosis and targeted treatment of neurodegenerative disorders.

Despite these merits, protein-tagged photoactivatable systems are still in their infancy of research. Three critical research directions for future developments include the following. (1) Structural optimization: Rational design of protein-taggable photocages to enhance the quantum yield of uncaging, improve the specificity of protein tagging, and further redshift absorption into the second near-infrared (NIR-II) optical window. (2) Biological validation: Conduction of expanded in vivo studies using animal models to evaluate the feasibility of protein-taggable photocages. (3) Advancing precision and stability: The incorporation of protein-taggable photocages into smart delivery systems, such as polymers, lipids, exosomes, and organic–inorganic nanoparticles, to mitigate the off-target release and enhance long-term stability in vivo. By addressing these challenges, next-generation protein-taggable photocages could unlock transformative applications in various neurotheranostics and precision medicine.
